# A rare case of ventricular tachycardia caused by an intramyocardial fibroma with successful surgical resection in an adult

**DOI:** 10.1186/s13019-024-02986-3

**Published:** 2024-09-05

**Authors:** M. Scott Binder, Matthew Roby, William Chancellor, Leora Yarboro, Scott Arnold, Kyle Hodge, John Kern, David Sane

**Affiliations:** 1 Department of Cardiology, Virginia Tech Carilion Roanoke Memorial Hospital, 1906 Belleview Ave SE, Roanoke, VA 24015 USA; 2 Department of Cardiothoracic Surgery, University of Virginia, Charlottesville, VA USA; 3 Department of Cardiothoracic Surgery, Virginia Tech Carilion Roanoke Memorial Hospital, Roanoke, VA USA; 4 Department of Pathology, University of Virginia, Charlottesville, VA USA

**Keywords:** Cardiac fibroma, Cardiothoracic surgery, Multimodality imaging, Ventricular tachycardia

## Abstract

**Background:**

Cardiac fibromas are extremely rare in adults. The preferred treatment is surgical resection, but antiarrhythmic medications or heart transplantation have also been used previously. The cardiac imaging, particularly MRI, can be useful to help delineate between primary cardiac tumors, and surgical factors such as the extent/size of the fibroma, involvement of the coronary arteries or mitral apparatus and amount of residual myocardium influence whether surgical resection is feasible.

**Case presentation:**

A 42-year-old male presented with a wide-complex tachycardia, unresponsive to amiodarone. An echocardiogram was performed which showed a possible posterior wall mass. A cardiac MRI showed a well circumscribed lateral wall intracardiac fibroma, measuring 5.2 × 5.1 × 3.8 cm with preserved function. Surgical resection was successful, and he was discharged without a defibrillator.

**Conclusions:**

Cardiac fibromas are encapsulated tumors which do not infiltrate myocardium and should be surgically resected if possible.

**Supplementary Information:**

The online version contains supplementary material available at 10.1186/s13019-024-02986-3.

## Background

Cardiac fibromas in adults are extremely rare, and there are no consensus guidelines regarding management. Case reports have recommended surgical resection when feasible, but other cases have used antiarrhythmic medications or heart transplant as alternative treatment options. There is very little information regarding the optimal surgical approach and when or if patients are surgical candidates for resection. Operative treatment recommendations have typically been based upon the experience of the surgeon performing the procedure.

## Case presentation

**Fig. 1:**
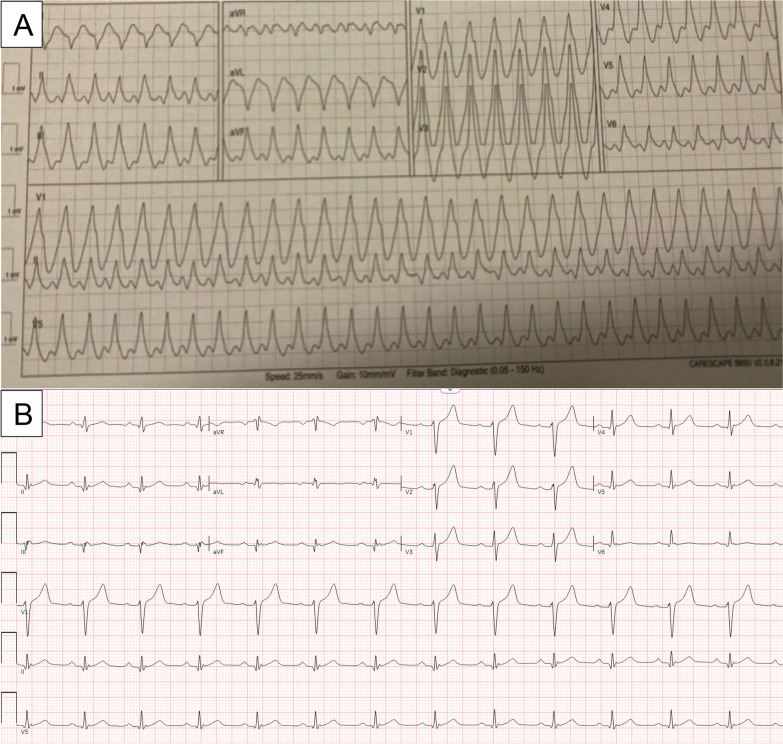
**A**. ECG in Wide Complex Tachycardia. Panel A illustrates his wide complex tachycardia at a rate of around 220 beats per minute. Positive inferior leads (II, III, aVF) indicate a basal/mid ventricular or outflow tract origin, and positive concordance throughout the precordial leads suggests a posterior ventricular origin and argues against a supraventricular tachycardia with aberrancy. **B**. Baseline ECG. Panel B illustrates his baseline ECG, which demonstrates nonspecific T wave flattening, a normal axis and notable lack of delta waves indicative of pre-excitation

**Fig. 2:**
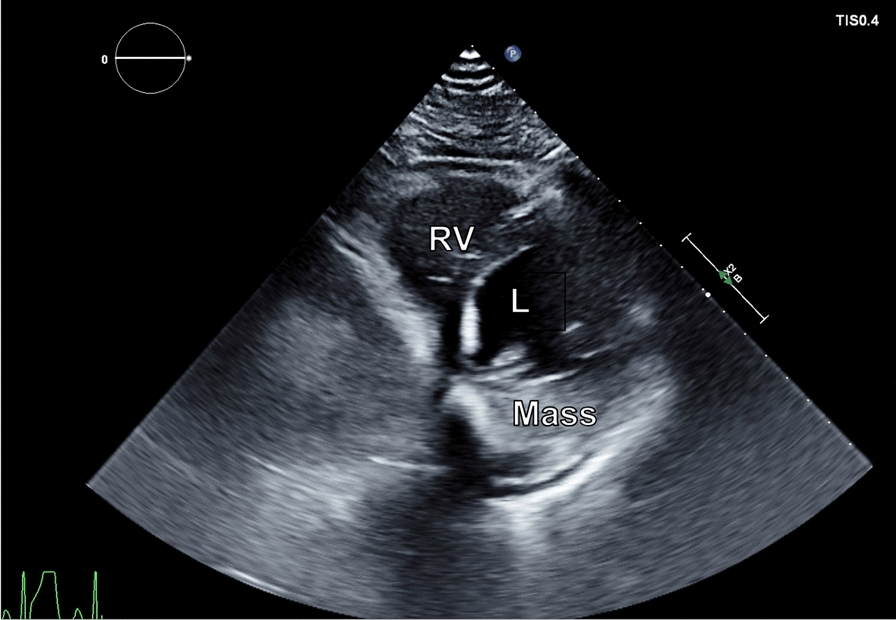
Transthoracic Echocardiogram: A transthoracic echocardiogram illustrates a large mass in the posterior wall of the left ventricle (LV) with normal right ventricular (RV) size

A 42-year-old male was transferred from an outside hospital for a wide complex tachycardia and concern for left ventricular mass. His ECG is shown (Fig. 1) and was concerning for a rapid VT (around 220 beats per minute), given the width of the QRS complex and positive concordance throughout the precordial leads. He was started on Amiodarone and underwent synchronized cardioversion, but by report his rhythm degenerated into VF and he underwent CPR for 1 min before his rhythm stabilized. He was switched to procainamide and admitted to the hospital. He underwent a TTE which illustrated a large basal to mid posterior wall mass (Fig. 2). He had a past medical history of potential Wolff-Parkinson-White (WPW) syndrome (following a wide complex arrhythmia at age 17, declined electrophysiologic study at that time). He also had a history of hypertension, hyperlipidemia, and type 1 diabetes on insulin.

The differential diagnosis was broad, and including benign (cardiac fibroma, rhabdomyoma, myxoma, hemangioma, or lipoma) or malignant/metastatic etiologies for his mass which could result in his VT. Additionally, an atrioventricular re-entrant tachycardia was considered, given his potential history of WPW, but his baseline ECG lacked delta waves and no pre-excitation was visualized on telemetry (Fig. 1). Outflow tract or bundle branch re-entrant tachycardias were also considered, but less likely given the morphology of the VT.

**Fig. 3:**
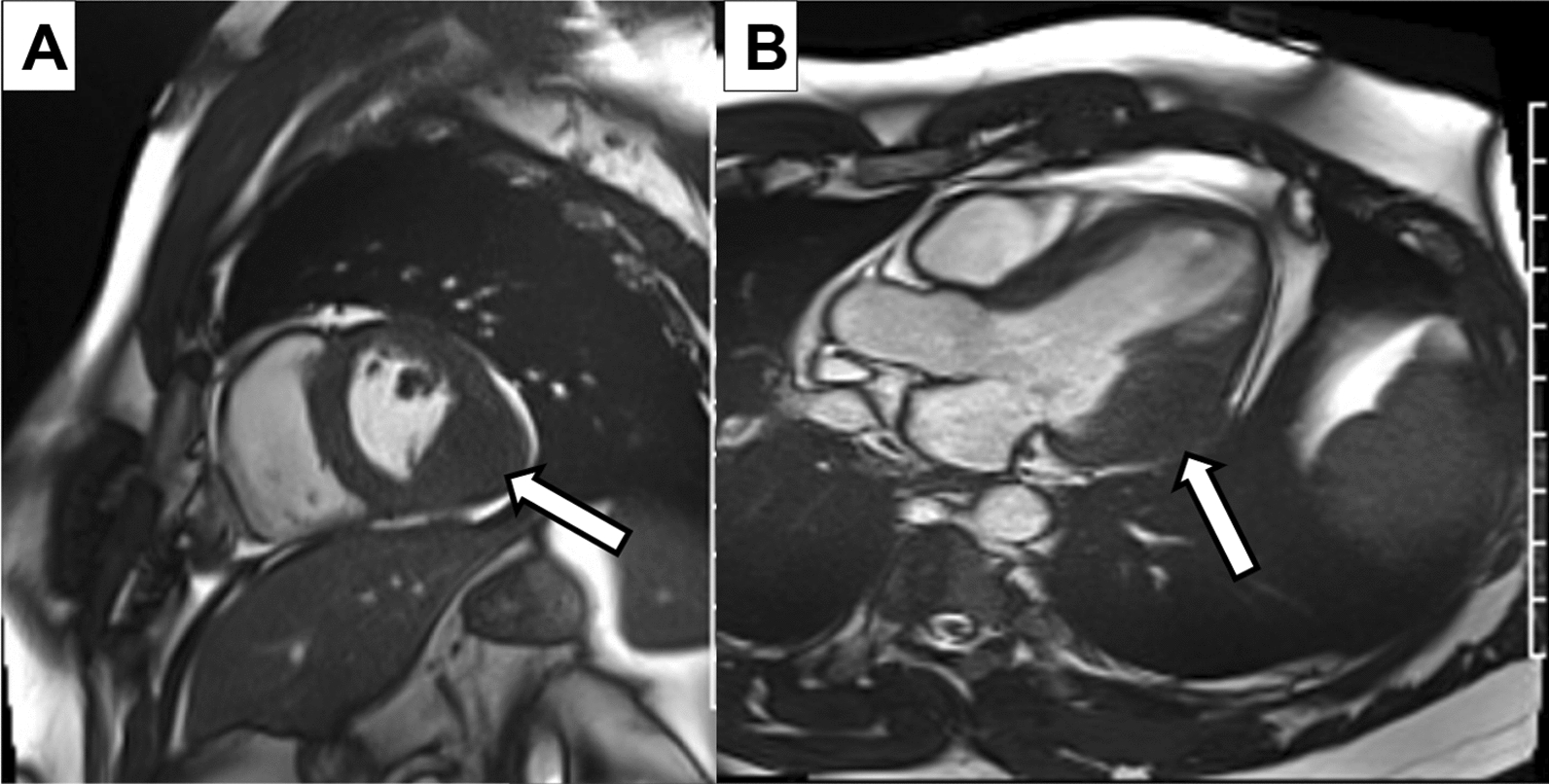
Cardiac MRI: A short axis basal section **A** and apical 3 chamber **B** on cardiac MRI illustrates the large inferolateral/posterior wall mass (arrows) which is well circumscribed and iso-intense to native myocardium

**Fig. 4:**
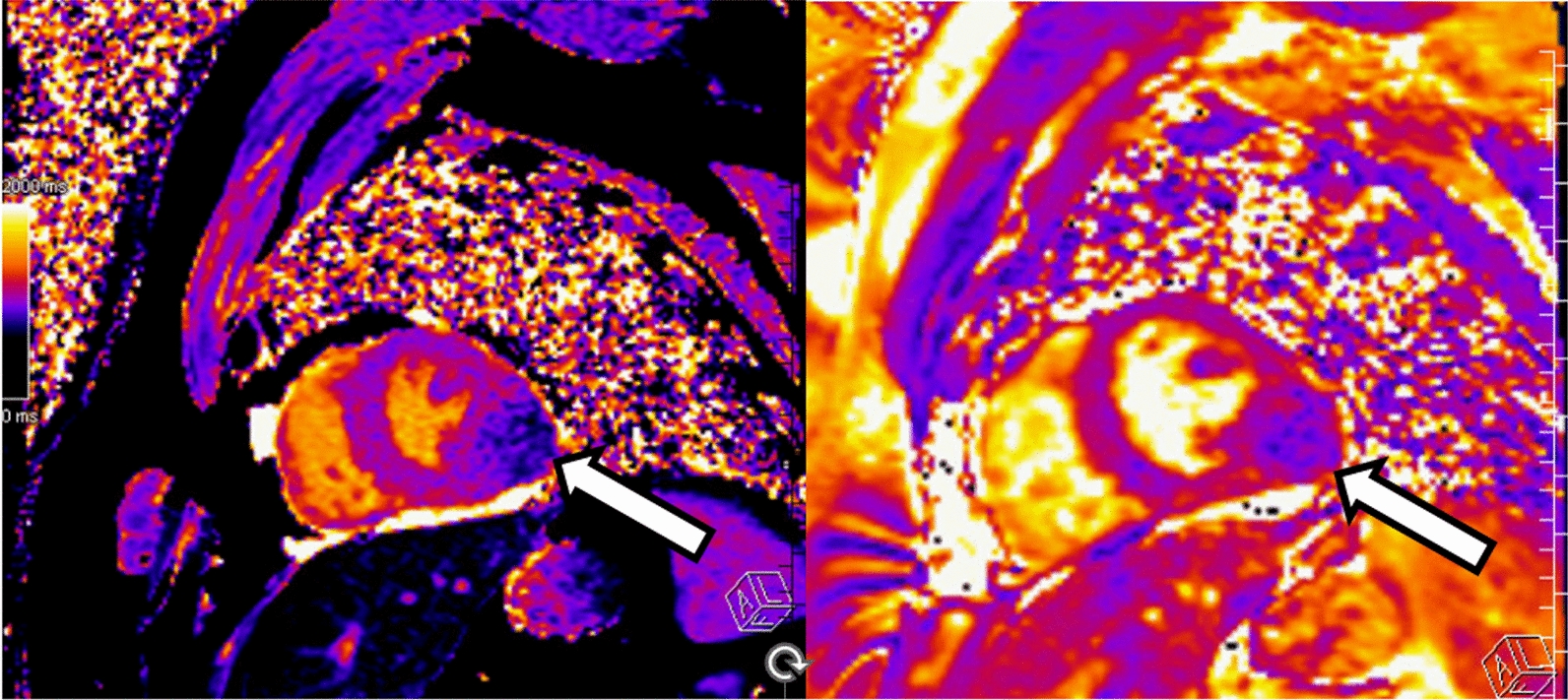
T1 and T2 Maps: Shown are the T1 (left) and T2 (right) maps of short axis sections on the cardiac MRI. The mass (arrows) is hypointense on T1 and T2 weighted imaging relative to native myocardium

**Fig. 5:**
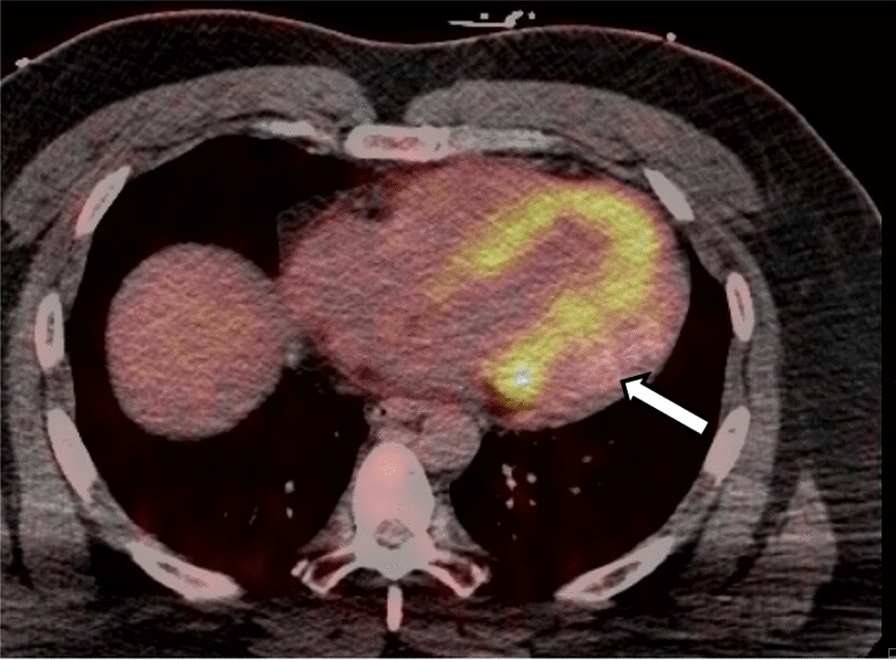
FDG-PET Scan: An FDG-PET axial slice at the level of the left ventricle, which illustrates hypointense uptake of the mass (arrow) relative to native myocardium. There is no significant uptake of FDG elsewhere in the heart

A cardiac MRI was obtained for further tissue characterization of the mass. It revealed a large, well circumscribed mass in the basal to mid lateral wall, which measured 5.2 × 5.1 × 3.8 cm (Fig. 3). Left and right ventricular function was preserved. There was poor uptake on first pass perfusion, and tissue mapping showed low T1 and T2 values for the mass, relative to native myocardium (Fig. 4). An FDG-PET was obtained to evaluate for metabolic activity of the mass and any concurrent masses or metastases. It showed hypointense uptake of the mass relative to native myocardium (Fig. 5) and no significant extra-cardiac uptake. A CCT was also obtained which showed no significant coronary involvement and no coronary calcification or stenosis. Given the hypointense uptake of the mass, low T1 and T2 values and association with VT, a working diagnosis of an intramyocardial fibroma was made.

**Fig. 6:**
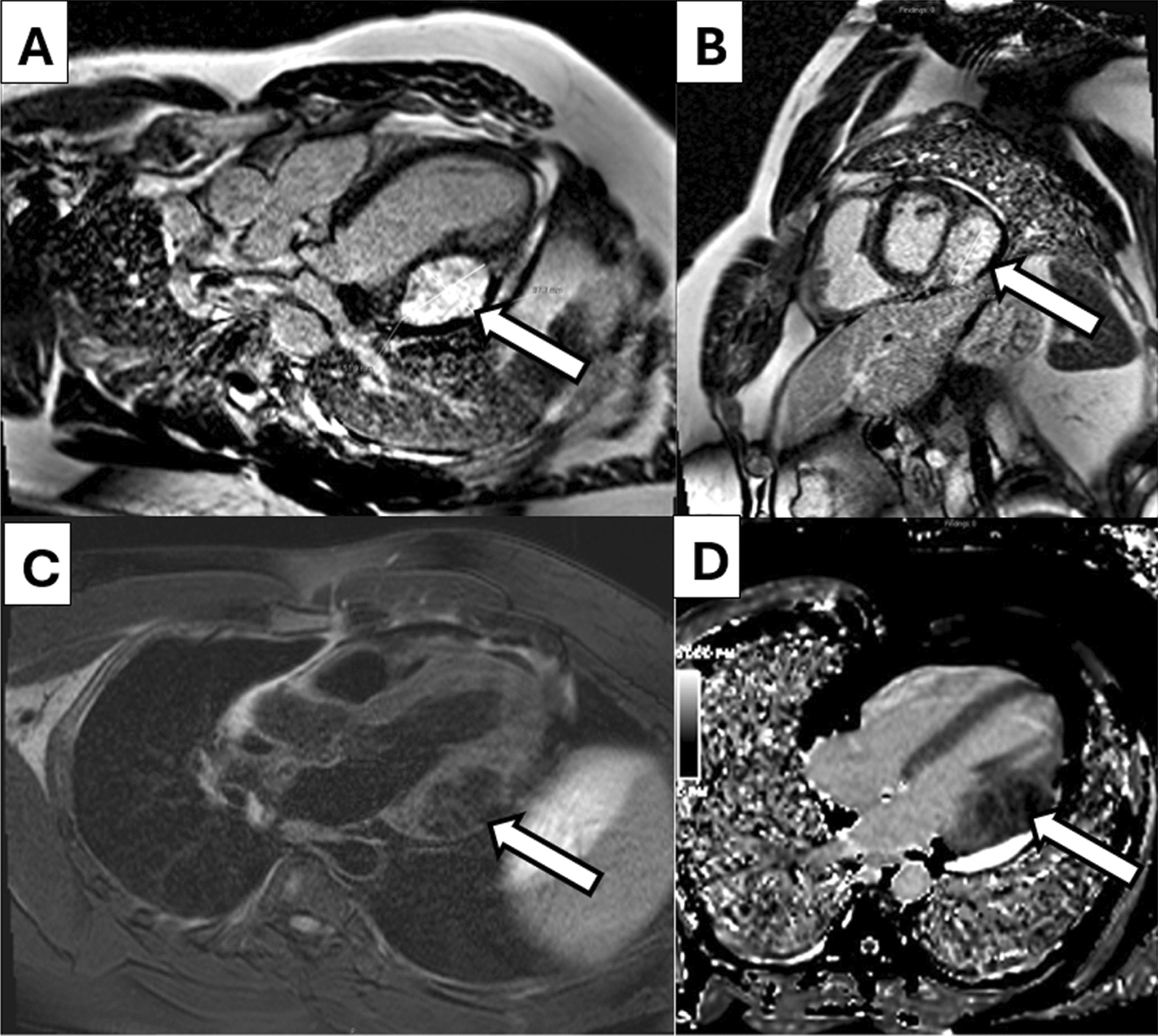
Repeat MRI Images: A repeat MRI was obtained for sizing and evaluation of the mass with the mitral valve apparatus. On late gadolinium enhancement (LGE) images, the mass (arrows) has extensive LGE avidity **A**, **B** and measured 5.2 × 5.1 × 3.8 cm. On T1 **C** and T2 **D** weighted imaging, there did not appear to be involvement of the mitral valve appartatus and the mass appeared well circumscribed compared to native myocardium

A repeat MRI was obtained to ensure the mass did not infiltrate the atrioventricular groove or interfere with the mitral valve apparatus or papillary muscles. The MRI did not illustrate any involvement of the atrioventricular grove or mitral apparatus and did not appear to enter the cavity of the left ventricle (Fig. 6A–D). Given the localization of the mass without involvement of surrounding critical structures, surgical resection was recommended.

**Fig. 7:**
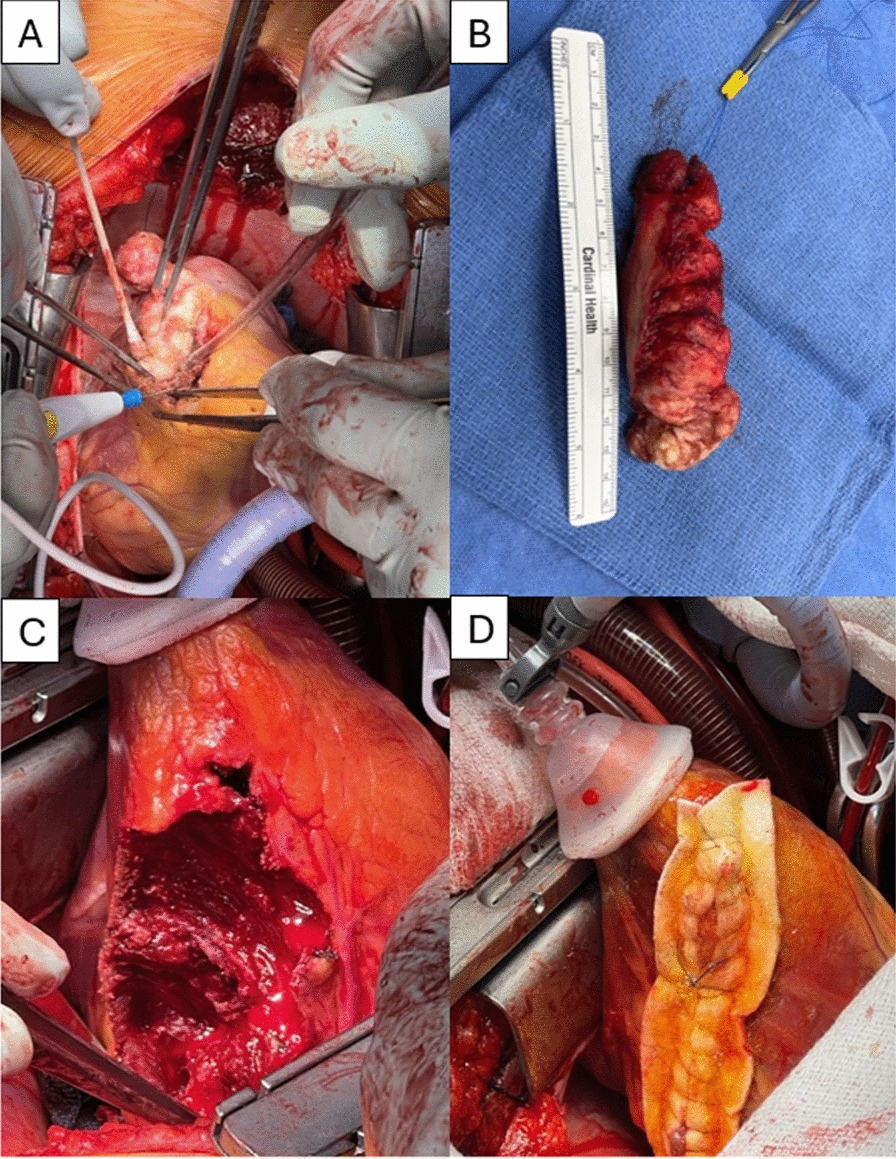
Gross Specimens: Panel** A** illustrates the fibroma removal from the lateral wall of the left ventricle. Panel** B** shows the final specimen after removal, around 10 cm in length. Panel** C** is the residual ventricular myocardium, without entry into the lumen, which was sutured closed over felt (panel** D**)

**Fig. 8:**
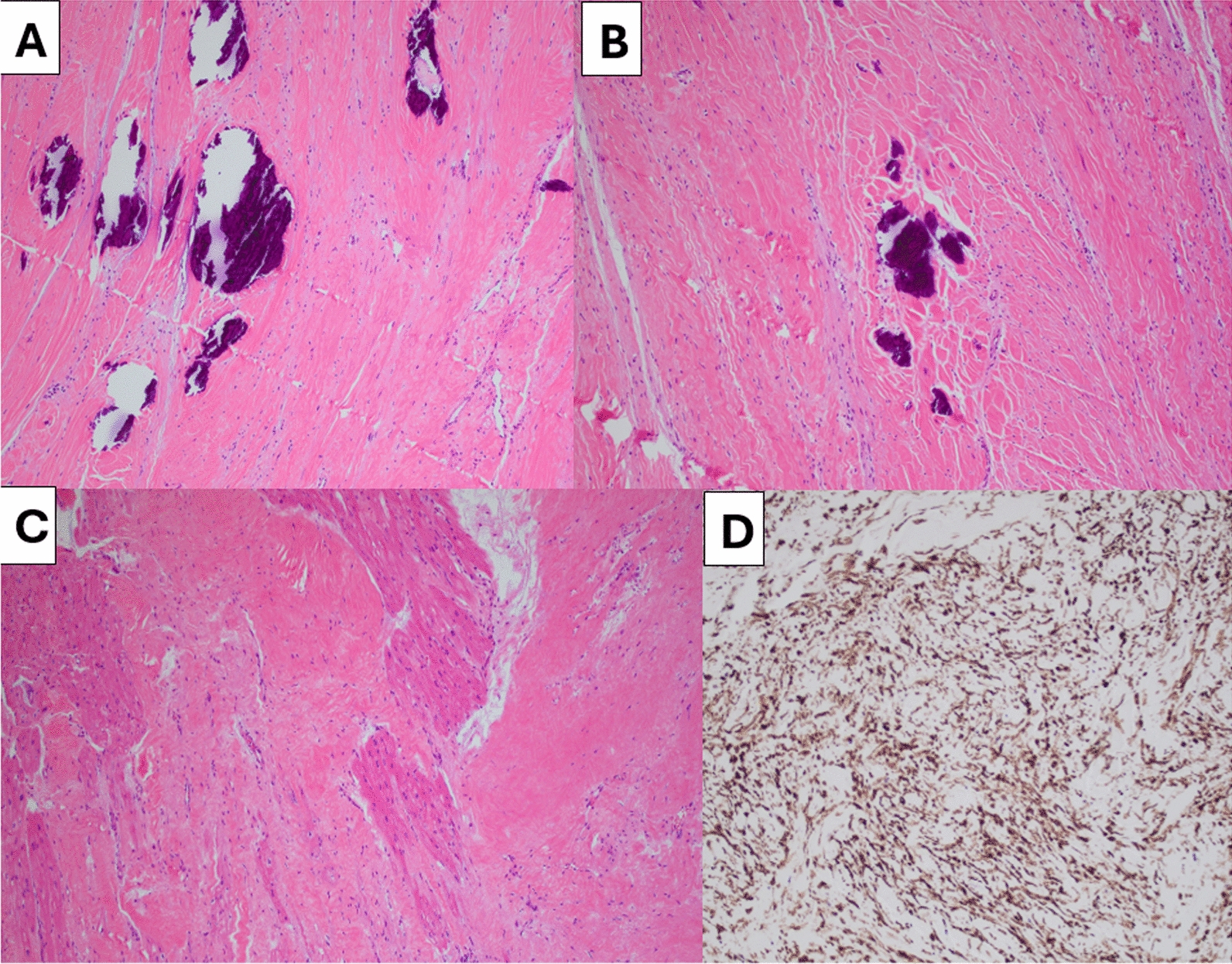
Pathologic Specimens: Histologic sections show bland spindle cells in irregular, loose fascicles within collagenous stroma. Linear cutting artefact is identified secondary to calcifications. **A**–**B**. Tumor cells can be seen juxtaposed with uninvolved myocardium **C**. The tumor cells strongly express SMA by immunohistochemical staining **D**

The patient underwent a median sternotomy, followed by anterograde and retrograde cardioplegia and root ventilation with cardiopulmonary bypass initiation. The heart was lifted out of the mediastinum with an Octopus tissue stabilizer, so that the posterior wall of the heart could be visualized (Fig. 6). A firm mass was palpated, and the mass was dissected away from the apical myocardium using sharp dissection and electrocautery. The mass did not violate the endocardium. Terminal branches of the coronary arteries were ligated when required. Following cardiac mass removal, there was a 10 cm by 4 cm defect in the posterior wall which was closed with primary edge-to-edge repair over 2 large felt strips and reinforced with bioglue (Fig. 6). The total cardiopulmonary bypass time was 99 min, and the cross-clamp time was 79 min. An advanced heart failure cardiothoracic surgeon was also present during the procedure, in case the need arose for a left ventricular assist device or total artificial heart following mass resection. Pathology was consistent with an intramyocardial fibroma with calcifications and positive SMA staining (Fig. 7A–D). He had no recurrent sustained ventricular arrhythmias while hospitalized and was discharged on amiodarone and had a 30-day monitor placed to monitor for recurrences (Fig. 8).

## Discussion

Cardiac fibromas and rhabdomyomas are the most common benign tumors in children, but newly diagnosed fibromas in adulthood are extremely rare [1]. Most patients with fibromas are asymptomatic and discovered incidentally, but there are associations with ventricular arrhythmias, heart failure or sudden cardiac death [1]. Notably, unlike rhabdomyomas, fibromas do not regress over time [2]. The mass may grow until the heart reaches mature size, usually around 17–20 years of age, and then remain stable, but the relative size of the fibroma to cardiac mass decreases [3]. Retrospective studies have found that most adult patients presented with palpitations (31%) or syncope (15%), commonly due to ventricular tachycardia, with heart failure (12%) and anginal symptoms (15%) occurring less frequently [4]. Younger patients have a smaller heart, so tend to present with heart failure due to larger tumor to heart ratios, whereas older patients present with ventricular arrhythmias most commonly [3].

Given its rarity, there are no standardized guidelines for management of fibromas, but options include surgical resection, medical management (typically with beta blockers and amiodarone) or heart transplantation with or without the need for single ventricle palliation as a bridge have also been reported [3–6]. Given the benign nature of cardiac fibromas, chemotherapy and radiotherapy are not standard treatment options, although they have been effective in primary malignant tumors of the heart prior to resection, such as sarcomas [7]. If patients are surgical candidates, this is generally the preferred approach, and a median survival of 27 years following successful resection has been reported in retrospective studies [3, 8]. Younger age at diagnosis has been found to be a poor prognostic indicator, likely due to the larger relative tumor size compared to cardiac mass [9]. Additionally, tumor location in the interventricular septum is associated with significantly increased mortality, thought to be due to involvement of the conduction system and predilection for increased arrhythmias [9]. Occasionally, complex operations including pericardial or synthetic patches, valve replacement or bypass grafting are required, depending on the extent of tumor involvement [10]. Surgical resection is associated with excellent long-term outcomes, up to 30 years post-operatively [11]. Subtotal resection is an option if tumor extent involves critical structures, and there was not a change in size for the residual tumor, although sample size was small [11]. There can also be misdiagnosis of primary cardiac masses with cardiac imaging, making histopathologic correlation crucial to confirm the diagnosis and benign or malignant nature of the mass [12].

It is likely that his wide complex tachycardia at the age of 17 was misdiagnosed as WPW syndrome as well and was the first manifestation of his cardiac mass, although the details of this arrhythmia and strips are not available from that time.

The patient is doing well at six-month follow-up and has not had any recurrences of his ventricular arrhythmias. His cardiac monitor did not show any atrial or ventricular arrhythmias over 30 days. His ventricular function remains preserved following surgery and he has been weaned off amiodarone in the outpatient setting. He has no activity restrictions following his procedure. There are no plans for ICD placement currently. He had a repeat cardiac MRI which showed a preserved EF (56%) without any notable mitral regurgitation. He was re-admitted for 1 day 3 months after mass removal for a small pericardial effusion and pleuritic chest pain, thought to be from pericarditis. He had a repeat transthoracic echocardiogram (video 1, 2) which showed a preserved EF (55–60%) and hypokinesis of the inferolateral and anterolateral wall segments, with a small pericardial effusion.

## Conclusions

An intramyocardial fibroma is an extremely rare cause of VT in adults, and several treatment options exist. For patients who are surgical candidates, resection is recommended to help prevent growth and recurrence of arrhythmias.

## Supplementary Information

Video 1: Post-Operative Transthoracic Echocardiogram: A transthoracic echocardiogram at the mid-short axis level using Definity contrast, which illustrates a preserved ejection fraction with hypokinesis of the inferolateral wall following fibroma removal.

Video 2: Post-Operative Transthoracic Echocardiogram: A transthoracic echocardiogram in the apical-4-chamber orientation with color Doppler, which illustrates no significant mitral regurgitation and hypokinesis of the anterolateral wall.

## Data Availability

Not applicable.

## Abbreviations

CCT: Cardiac computed tomography
cMRI: Cardiac magnetic resonance imaging
CPR: Cardiopulmonary resuscitation
FDG-PET: Fluorodeoxyglucose- positron emitted tomography
TTE: Transthoracic echocardiogram
VF: Ventricular fibrillation
VT: Ventricular tachycardia
